# Gene Therapy in the Management of Erectile Dysfunction (ED): Past, Present, and Future

**DOI:** 10.1100/tsw.2009.102

**Published:** 2009-08-31

**Authors:** Arnold Melman, Kelvin P. Davies

**Affiliations:** Department of Urology, Albert Einstein College of Medicine, Bronx, NY, USA

**Keywords:** erectile dysfunction, gene transfer, potassium channels, Maxi-K, stem cells

## Abstract

In the past, many researchers considered viral vectors to be the most promising candidates to transfer genetic material into the corpora for the treatment of erectile dysfunction. However, at present, no viral vectors have progressed to human trials. In contrast, the use of naked gene therapy, a plasmid expressing the human Maxi-K potassium channel, is the only gene therapy treatment to be evaluated in clinical phase I trials to date. The success of these studies, proving the safety of this treatment, has paved the way for the development of future gene transfer techniques based on similar transfer methods, as well as novel treatment vectors, such as stem cell transfer.

## PAST

Human gene therapy is a novel, promising step towards the prevention and treatment of disease[1]. Of primary significance is tailoring the method of delivery of a gene of interest to the disease to be treated. Gene transfer to treat malignancies requires a high rate of efficiency of gene penetration and long-term gene expression so that all cells of a cancer will be altered and affected. For this purpose, viral vectors are needed. The target gene is packaged into a viral vector so that the gene can survive without enzymatic breakdown in the body fluids and cytoplasm of the target cells, and be persistently expressed from the nucleoplasm[2,3,4].

Smooth muscle disorders of the genitourinary system, such as overactive bladder (OAB) syndrome and erectile dysfunction (ED), share important differences from malignancies or extensive genetic disorders that make them attractive targets for gene therapy and, in particular, allow the use of naked DNA rather than viral vectors to effect cell membrane penetration with the gene of interest. The therapeutic goal of therapy in both ED and OAB is modulation of smooth muscle tone to cause an organ with heightened tone to be relaxed rather than to alter structure, function, or initiate apoptosis[5,6,7,8,9]. Additionally, the organs of the genitourinary system are readily accessible and available for local delivery of the gene product. Therefore, the possibility of untoward events from random, nonspecific biodistribution into unintended sites is minimized[2].

As indicated in the Fig. 1 legend, the use of naked DNA, while having the advantage of safety because of its lack of chromosomal integration, also has the disadvantage of being highly susceptible to breakdown by endonucleases that render it ineffective. Thus, one of the primary goals for the use of naked DNA in both

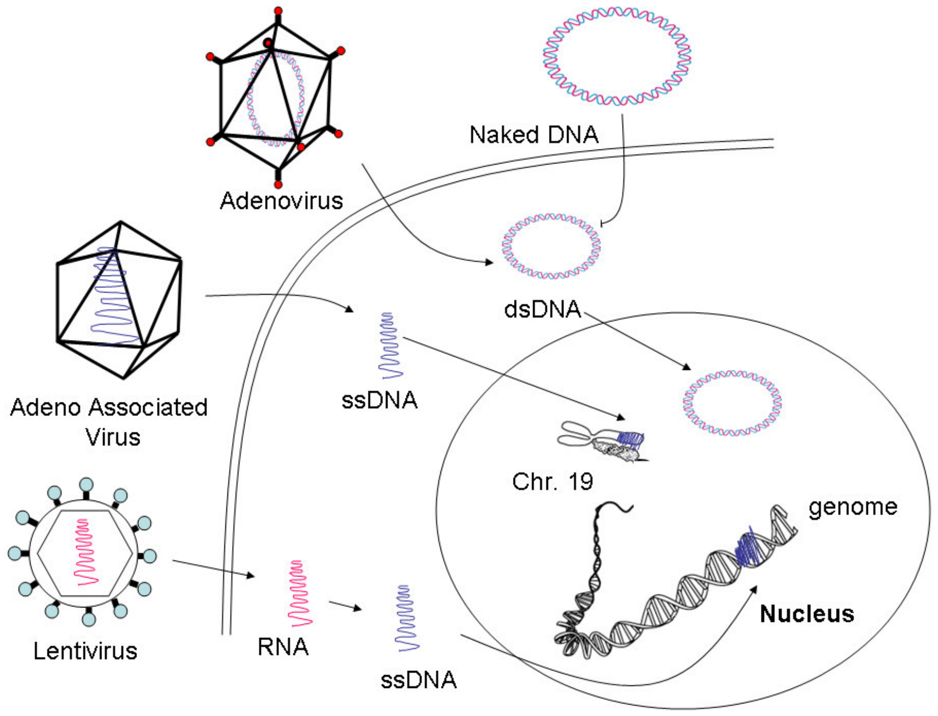

**FIGURE 1.** The differences in the cellular effect of viral vs. naked DNA vectors. Note that after transfer with a lentivirus vector, the gene is permanently integrated into a chromosome and is replicated. Another viral vector proposed for gene therapy is the adeno-associated virus, which integrates genetic material specifically into chromosome 19. When the adenoviral vector is used for gene therapy, the genetic material from the virus is not integrated, but resides in the nucleoplasm and is replicated. After naked DNA transfer, the plasmid resides in the nucleoplasm, but is not replicated.

preclinical trials in animal models as well as human trials is to determine the duration of expression (in animal models) and of the physiological effect in the human trials.

Erection is an event that requires proper function of arterial inflow, relaxation of corporal smooth muscle, and occlusion of out-flowing blood to achieve a sustained, long-lasting, rigid erection sufficient for sexual satisfaction[10,11,12]. The most commonly used therapy for the treatment of most cases of ED is with oral phosphodiesterase inhibitors.

Although the current generations of oral phosphodiesterase-5 (PDE-5) inhibitors for the treatment of ED are now a standard first-line therapy for the treatment of ED, there are problems with their use. Cost is a major issue for many men, particularly during this time of fiscal crisis and insurance company cutbacks that have diminished, or totally eliminated, the number of pills paid for per month. The PDE-5s are least effective in men who have more severe ED, including men who have diabetes, and are contraindicated in association with some families of drugs or medical conditions. The need for on-demand ingestion of the drug limits the spontaneity of the sexual act, leading to the need for “planned sex”. There is also a well-described number of side effects, compounded by effects on the absorption and pharmacokinetics due to diet.

Several investigative laboratories have approached the potential use of gene therapy for ED by employing a variety of vectors to express target genes in the corporal bodies, primarily in rodent models of ED (Table 1). Interestingly, the majority of these studies demonstrated improved erections and cavernous pressures in response to cavernous nerve neurostimulation in the rat, demonstrating the plasticity of the erectile apparatus. However, the inherent immunogenicity of the viral vectors has limited the improvement of erectile function to weeks in the rodent model, and the use of viral vectors in humans has risks of a lethally pathologic immune response.

**TABLE 1**
**Comparison of Gene Therapy Studies for ED**

| Authors, Year, Ref. | Vector | Gene | Animal Model | Duration |
|---|---|---|---|---|
| Rogers et al. (2003)[13] | 1. Recombinant VEGF protein<br>2. AAV<br>3. AAV-LacZ | VEGF | Male Sprague-Dawley rats, 3–6 mo old (350–450 g) | 1 mo after administration |
| Chancellor et al. (2003)[14] | 1. Plasmid<br>2. Adenovirus<br>3. Adenovirus-transduced myoblast cells | 1. iNOS | Adult male Sprague-Dawley rats (250–400 g); no disease | 2, 4, or 7 d after administration |
| Champion et al. (1999)[15] | 1. Replication-deficient recombinant adenovirus | 1. eNOs gene (AdCMVeNOS)<br>2. Stereotype 5-encoding nuclear-targeted beta-galactosidase (AdCMVbeta-gal) | Male Sprague–Dawley rats, 40 weeks old (350–400 g); no disease | 1 d after adenovirus administration |
| Garban et al. (1997)[16] | 1. Plasmid (exogenous iNOS cDNA contracts) | 1. iNOS inducers | Male Fischer 344 rats, 5, 20, and 30 mo old | 3–21 d after administration |
| Magee et al. (2002)[17] | 1. Plasmid cDNA construct (pCMV-PnNOS)<br>2. Helper-dependent adenovirus (Adv-CMV-PnNOS) | 1. beta-Galactosidase<br>2. PnNOS | Male Fischer 344 rats: young rats (5 mo old), aged rats (24 mo old), and retired breeders (9–11 mo old) were used | 1. Assessment of beta-galactosidase expression was estimated 11 d after administration<br>2. After 18 d for PnNOS administration |
| Bivalacqua et al. (2001)[18] | 1. Adenoviral-mediated transfer (AdRSVCGRP)<br>2. Adenovirus (AdRSVbetagal) | 1. Prepo-CGRP<br>2. Nuclear-targeted beta-galactosidase | Brown Norway rats, 12 (225–300 g) and 60 weeks (450–550 g) old | 5 d after administration |
| Bivalacqua et al. (2003)[19,20] | 1. Adenovirus (AdCMVbeta-gal)<br>2. Adenovirus (AdCMVeNOS) | 1. beta-Galactosidase<br>2. eNOS | Total of 44 adult male CD rats divided into four groups | 1–2 d after administration |
| Bivalacqua et al. (2004)[21] | AAV | Dominant-negative RhoA (T19NRhoA) | STZ diabetic rats | 7 d after administration |
| Jin et al. (2006)[22] | AAV | Dominant-negative RhoA (T19NRhoA) | Young and old rats | 7 d after administration |
| Shen et al. (2005)[23] | pCDNA | VIP | 61 STZ-induced diabetic rats | 3, 7, and 14 d after administration |
| Magee et al. (2007)[24] | 1. Plasmid cDNA construct (pCMV-PnNOS) | (pCMV-PIN), pCMV-PIN antisense RNA, pSilencer2.1-U6-PIN-shRNA; and pSilencer2.1-U6-randomer-shRNA | Male Fischer 344 aged rats (24 mo old) | 1 mo later, assessment was done by measuring intracavernosal pressure increase |
| Wang et al. (2007)[25] | 1. Adenovirus | AdCMV-βgal or AdCMV-IGF-1 | STZ-injected adult male Sprague-Dawley rats | 1–2 d after transfection |
| Kato et al. (2009)[26] | HSV | 1. lacZ<br>2. NT3 | STZ male Sprague-Dawley rats (300–400 g) | Assessment was done 4 weeks postinjection |

*Note:* mo, months; d, day(s); AAV, adeno-associated virus; cGRP, calcitonin gene related peptide; HSV, herpes simplex virus; iNOS, inducible nitric oxide synthase; nNOS, neuronal nitric oxide synthase; STZ, streptozotocin; VEGF, vascular endothelial growth factor; VIP, vasoactive intestinal polypeptide.

Of greatest importance in a phase 1 clinical trial for a non-life-threatening illness is that the primary endpoint is safety not efficacy. The approach that we have taken at the Urology Laboratory at the Albert Einstein College of Medicine and through its academic-based company, Ion Channel Innovations, LLC, has been to use a plasmid-based vector for therapy of two smooth muscle disorders, ED and OAB, each of which has smooth muscle cell–heightened tone as an important aspect of the disease[5,9,27,28,29,30,31]. One of the genes that we have chosen to use for preclinical and clinical trials is KCNMA1, also referred to as hSlo, which encodes the alpha subunit of the potassium, large-conductance, calcium-activated channel, on chromosome 10; location 10q22.3. The channels are also known as Maxi-K and the product name we have chosen for our studies is *h*Maxi-K. Although the mature channel consists of a tetramer of alpha associated with beta subunits, *h*Maxi-K expresses only the alpha subunit. The alpha subunit forms a pore through which potassium ions can traverse the cytoplasmic membrane, whereas the beta subunit regulates the passage of potassium ions in response to intracellular calcium (Fig. 2).

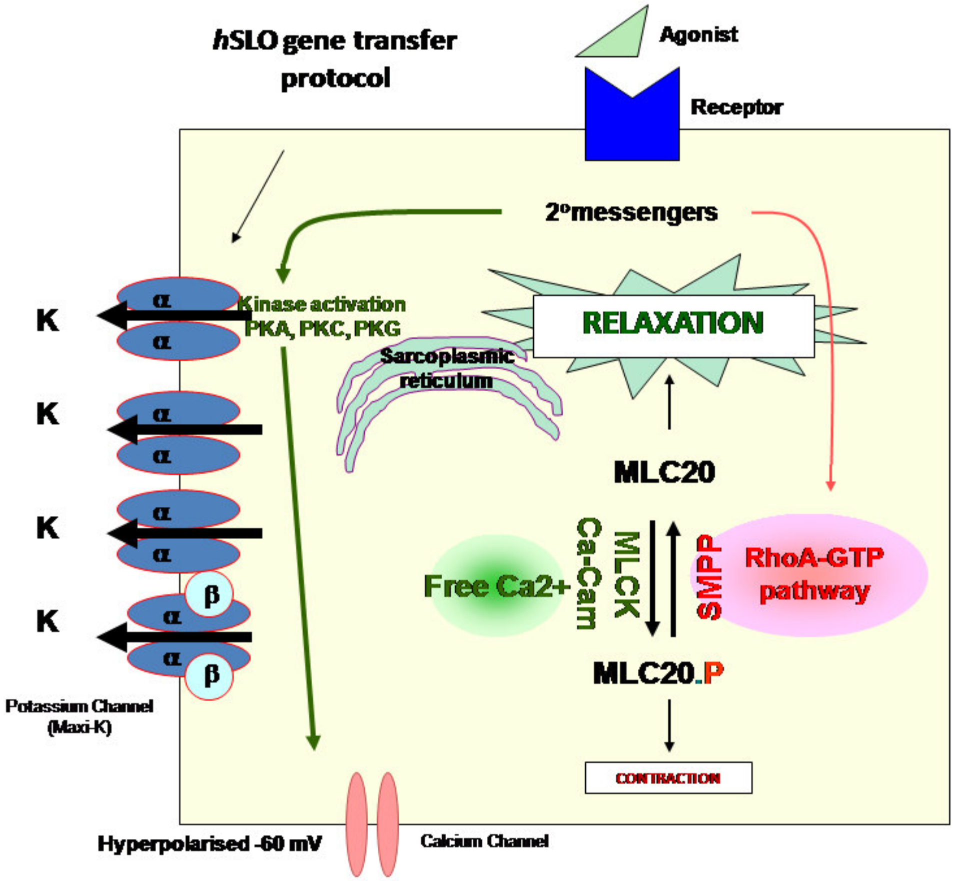

**FIGURE 2**. A representative smooth muscle cell that contains a native Maxi-K channel with natural expression of both an alpha and beta subunit, as well as a channel derived from *h*Maxi-K that expresses channels with only an alpha subunit. Both channels, when open, hyperpolarize the cell membrane and result in reduced calcium influx and reduction in smooth muscle cell tone.

In preclinical experiments, we have shown extensively that the physiological result of expressing the alpha subunit causes cell hyperpolarization, and smooth muscle cell and organ relaxation[7,27,30,32]. However, overexpressing only the alpha subunit channel appears to function within the complex network of pathways regulating smooth muscle tone as shown in Fig. 3.

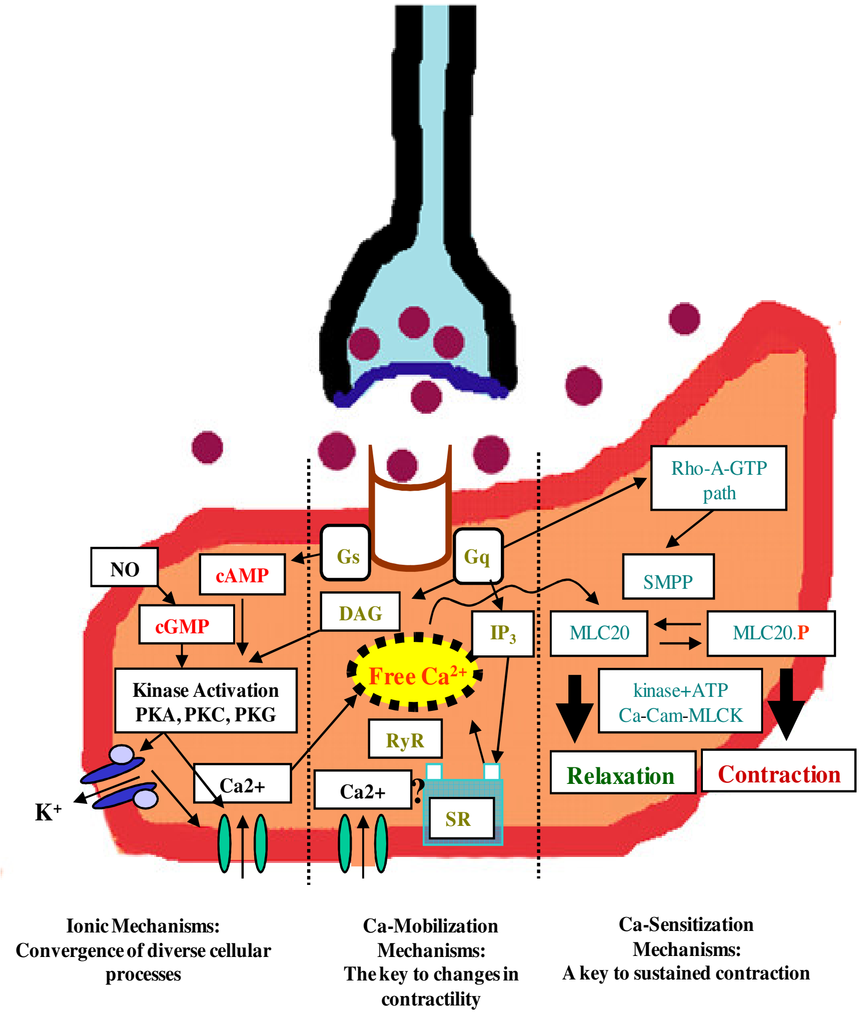

**FIGURE 3.** Three interdependent pathways in the smooth muscle cell that effect the contractile-relaxation response. The left panel shows the effect of both nitric oxides (NO) on potassium channels and calcium influx. The center panel shows determinants of intracellular free calcium. The right panel shows the interaction of the contractile proteins and the influence of RhoA on sustained calcium contraction.

## PRESENT

To date, we have completed a phase 1a and 1b clinical trial with *h*Maxi-K. The trial was an open-label, sequential, single-dose, intracavernous instillation of seven doses of *h*Maxi-K, 500–16,000 µg, in 21 men with moderate to severe ED as defined by International Index of Erectile Function (IIEF) score[9,28,29]. The specifics of the entire trial are presented elsewhere, but the most pertinent findings are listed below.

1. There were no clinically relevant, transfer-related, serious adverse events in any participant at any time up to 4 years after transfer.
2. There was no plasmid evident in the semen of the participants.
3. There were no clinically relevant changes in any of the physical or chemical parameters measured, including EKG.

4. In the men who responded with improved erections after transfer, the response lasted for greater than 6 months. This supports the preclinical trials in aged or diabetic male rats where efficacy was noted for periods of at least 6 months

## FUTURE

At the present time, with the exception of the trial noted above, there are no other FDA-approved, open ED trials that use gene transfer for the treatment of ED. Ion Channel Innovations, LLC, based on the positive results of the phase I trial, is planning to extend to a phase 2 trial that will include a placebo arm and an increased number of participants.

Ion Channel Innovations, LLC–sponsored research has resulted in two published[30,33], preclinical papers in which the structure of the backbone of the vector of the parent plasmid has been modified so that the Maxi-K gene is expressed specifically in smooth muscle cells. Studies — one in aging rats and the other in atherosclerotic, crab-eating monkeys with ED — were successful in normalizing erectile function. The advantage of a smooth muscle–specific promoter of gene expression is an enhanced safety profile, limiting expression to the target cells (smooth muscle cells) even when there is unintended biodistribution into potentially susceptible organs.

Further advances in gene transfer with either of the plasmid vectors will be directed to increase the transfection efficiency and duration of response following transfer[34,35,36,37,38].

In order to regain acceptance of viral vectors for clinical application, it will be necessary to improve their safety profiles. Investigators are working to reduce the immunogenicity and replication of adenoviral vectors, and prevent the possibility of insertional mutagenesis (potentially activating oncogenes) by retroviral vectors[37,39]. Another potential line of therapy is the use of small interfering RNA (siRNA), using either naked plasmid or retroviral vectors for reduction of gene expression[24,40].

An alternative to gene therapy, which is presently being evaluated by several research groups, is the use of adult stem cells derived from bone marrow, blood, or organ of interest, and instilled into various organs. The goal would be to replace defective or missing cells from the organ of interest. Thus, the stem cells would *transdifferentiate* into the cell of interest (see http://stemcells.nih.gov/info/basics4.asp for a stem cell primer). For example, Song et al. transferred immortalized human fetus–derived mesenchymal stem cells into rat corpora, which differentiated into both endothelial and smooth muscle cells[41]. Another future possibility, if the high expectations for stem cell utilization are found to be successful, is to combine gene(s) of interest and stem cells so that the gene of choice would be engineered *ex vivo* into the stem cell for targeted protein expression in the organs of choice[42,43,44,45,46,47]. The endothelium as the tissue of choice for therapy of ED has been explored by Bivalacqua et al.[42]. In those experiments, in aged rats over a short duration (21 days), the stem cells with and without the addition of an adenoviral vector containing bovine eNOS gene under control of a Rous sarcoma virus improved erectile function with the production of both calcium-dependent NOS activity and cGMP levels. Of course, the endothelium is only one potential tissue that can be altered with aging and disease. Smooth muscle cells, neurons, and small arteries each are viable and potential targets for future research. Despite the present obstacles, gene transfer for treatment of ED represents an exciting potential leap for mankind into the next generation of modern medical treatment of the afflictions of aging and chronic disease in which urological laboratories are at the forefront of exploration.
